# Validation of the TransplantTrace cfDNA Kidney assay for measurement of donor-derived cell-free DNA in transplant recipients

**DOI:** 10.3389/fmed.2026.1880342

**Published:** 2026-08-18

**Authors:** Monika Berg, Kelly VanBemmel, Sara Klustner, Sofia Westerling, Linnea Pettersson, Emelie Foord, Tasha Kalista, Michael Uhlin

**Affiliations:** 1 Devyser AB, Stockholm, Sweden; 2 Devyser Genomic Laboratories (DGL), Roswell, GA, United States; 3 Department of Medicine, Karolinska Institutet, Stockholm, Sweden

**Keywords:** centralized, donor-derived cell-free DNA, kidney transplantation, next-generation sequencing, performance, rejection, validation

## Abstract

**Introduction:**

Donor-derived cell-free DNA (dd-cfDNA) has emerged as a promising non-invasive marker for assessing allograft status and guiding clinical management in transplant recipients. Its utility in kidney transplantation has repeatedly been demonstrated in large studies showing a strong association between elevated dd-cfDNA levels and allograft injury or rejection. This study evaluated the performance of a centralized next-generation sequencing (NGS)-based assay for measurement of dd-cfDNA in patients post-kidney transplantation.

**Methods:**

The TransplantTrace cfDNA Kidney assay utilizes 50 insertion–deletion (indel) markers to discriminate dd-cfDNA. Evaluation of analytical performance included determination of input requirements, analytical sensitivity and specificity, as well as accuracy and precision parameters. Diagnostic performance was evaluated in a retrospective cohort of 104 post-transplantation samples by comparing dd-cfDNA results with biopsy-confirmed rejection.

**Results:**

The assay required low DNA input (2 ng) and demonstrated high analytical sensitivity, with a verified limit of detection of 0.2% and limit of quantification of 0.3% dd-cfDNA. Analytical accuracy was excellent (*R*^2^ = 1.00), with high repeatability and reproducibility across the reportable range of 0.2–30% dd-cfDNA. In the clinical validation, the assay showed high concordance with biopsy-confirmed rejection and excellent discriminatory performance for differentiating active from non-active rejection (AUC of 0.980). At a 1% cut-off, the assay exhibited a positive predictive value of 100%, supporting confident identification of patients likely to have treatable graft injury, while a high negative predictive value (95.6% at 15% prevalence) supports its reliability in ruling out active rejection.

**Conclusion:**

The TransplantTrace cfDNA Kidney assay demonstrated robust analytical performance and strong clinical concordance with biopsy-confirmed rejection status. Its high diagnostic accuracy supports reliable identification and exclusion of active rejection, with the potential to reduce reliance on invasive biopsy procedures in patients with elevated serum creatinine but low dd-cfDNA levels. In summary, the findings of this study support the implementation and use of this centralized assay for measurement of dd-cfDNA in patients post-kidney transplantation.

## 1 Introduction

Kidney transplantation is the preferred treatment for patients with end-stage renal disease, offering significantly improved patient survival and quality of life compared to dialysis (1). Despite progress in the field, long-term allograft survival remains a major challenge (2). Recent reports indicate that 10–35% of kidney allografts fail within 5 years post-transplantation, highlighting the need for improved strategies to detect and manage allograft injury at an earlier stage (2, 3). As the number of kidney transplantations continues to rise in both the United States and Europe (1, 3), the development of reliable and non-invasive monitoring tools of graft status becomes increasingly important. Timely and accurate identification of allograft injury and rejection is essential for guiding effective therapeutic interventions and preserving long-term graft function and survival in these patients.

Current clinical strategies for monitoring kidney allograft function primarily rely on serum creatinine, estimated glomerular filtration rate (eGFR), measurement of proteinuria, and regular screening for donor-specific antibodies (DSA). However, these may be late or lagging indicators of kidney function and organ rejection. By the time these biomarkers are detected, irreversible allograft damage may have already occurred, increasing the risk of chronic graft dysfunction and loss (4). Surveillance protocol biopsies, or clinically indicated for-cause biopsies, are used to histologically evaluate allograft status and confirm or rule out rejection. However, biopsies are invasive, expensive, prone to sampling error, and are associated with risks such as bleeding, increased risk of opportunistic infections, and hospitalization (5).

Over the past decade, donor-derived cell-free DNA (dd-cfDNA) has emerged as a promising non-invasive biomarker for assessing allograft injury and rejection across various transplantation settings, including heart, liver and lung, with kidney transplantation being the most extensively studied (6). Pivotal studies have demonstrated that elevated levels of dd-cfDNA in kidney recipient plasma correlate with acute rejection and may reflect subclinical immune activity, supporting its utility and role in longitudinal surveillance and early intervention following kidney transplantation (7–10). The DART-study demonstrated that a dd-cfDNA threshold of 1% provided high diagnostic performance for antibody-mediated rejection (ABMR), with a negative predictive value (NPV) of 96%, outperforming serum creatinine, which failed to discriminate between rejection and non-rejection states (11, 12). The large-scale ADMIRAL-study, which prospectively followed over 1,000 transplant recipients, showed that dd-cfDNA levels ≥0.5% were associated with both clinical and subclinical rejection, tripled the risk of developing *de novo* (dn) DSA, preceded DSA identification by nearly 3 months, and predicted declines in eGFR (13).

Recent clinical studies have expanded the utility of dd-cfDNA across a range of clinical scenarios. A large multinational study by Aubert et al., involving more than 2,800 kidney recipients, showed that incorporating dd-cfDNA into clinical prediction models significantly improved the discrimination of rejection, including subclinical ABMR, T-cell mediated rejection (TCMR), and mixed rejections (14). Similarly, the TRIFECTA-study consistently supported the diagnostic accuracy of dd-cfDNA and demonstrated that low dd-cfDNA levels can reliably rule out rejection (15–17). Importantly, dd-cfDNA has shown utility even in histologically normal biopsies. Elevated dd-cfDNA levels identified molecular injury not seen by histology, suggesting that dd-cfDNA can detect early or subclinical pathology that would otherwise be missed by biopsy (18). The ProActive-study also demonstrated that dd-cfDNA level elevation could precede biopsy-confirmed ABMR by up to 5 months and well ahead of changes in serum creatinine. Even in patients with histologically normal biopsies, persistently elevated dd-cfDNA (≥1%) was associated with lower eGFR, suggesting ongoing, undetected graft injury (4). Additional insights from real-world settings reinforce the clinical relevance of dd-cfDNA. A study involving patients with sequential kidney transplants found that residual dd-cfDNA from a prior, non-functioning graft was still detectable in 20% of patients, highlighting the benefit of incorporating donor genotyping into dd-cfDNA workflows (19). Recently, Tian et al. explored the link between dd-cfDNA and dnDSA by demonstrating that dd-cfDNA levels were significantly higher in patients with “true” dnDSA (the true classified as determined by analyzing reactivity and HLA typing). This study indicated that dd-cfDNA dynamics can reflect evolving alloimmune activity and supported its use alongside dnDSA monitoring as a non-invasive, informative strategy for assessing graft injury and guiding clinical management (20).

The role of dd-cfDNA as a sensitive, non-invasive indicator of allograft status highlights its utility in kidney transplantation (21). In line with this development, the American Society of Transplant Surgeons updated its recommendations in 2024, advocating for dd-cfDNA testing in kidney transplant recipients with allograft dysfunction to rule out the presence of rejection. Currently in the field of dd-cfDNA, several commercially available centralized options have emerged for the detection of dd-cfDNA. Although these platforms have been instrumental in demonstrating the utility of dd-cfDNA in identifying allograft injury and informing clinical decision-making, opportunities remain to improve assay characteristics as well as facilitate and improve integration into routine clinical workflows. In parallel to these centralized assays, there have also been the emergence of decentralized assays (6, 22, 23).

In the current study, we aimed to conduct a comprehensive analytical and clinical validation of the centralized targeted NGS-based TransplantTrace cfDNA Kidney assay for detection of dd-cfDNA in kidney transplant recipients. The findings demonstrate a highly reliable and robust assay, providing sensitive and precise detection of dd-cfDNA with high diagnostic accuracy in detecting kidney allograft rejection. The results support the implementation of the assay into routine clinical practice, highlighting its favorable characteristics, for use as a non-invasive tool for monitoring kidney allograft health and guiding post-transplant clinical management.

## 2 Materials and methods

This study was designed to support a comprehensive evaluation of the TransplantTrace cfDNA Kidney assay performed at the CAP-accredited Devyser Genomic Laboratories (DGL) in Roswell, Georgia, USA. The samples consisted of simulated mixture samples and healthy blood donor samples for analytical validation studies, and clinical samples collected from kidney transplant recipients and their donors for the clinical validation study as further detailed in sections below.

### 2.1 Reference materials

Three dilution series were generated using a mixture of two gDNA samples with different genotypes (obtained from the Coriell Institute for Medical Research), totaling six genotypes in three simulated recipient-donor pairs. To simulate varying levels of dd-cfDNA, samples were sheared to mimic cfDNA fragment sizes (~166 bp) using the Bioruptor Pico (Diagenode). Fragmentation and concentration were verified using the TapeStation cfDNA ScreenTape (Agilent) and Qubit HS Assay (Thermo Fisher), respectively. Mixtures of the simulated donor-recipient pairs were generated with dd-cfDNA fractions ranging from 0.1 to 30%, and used in assessments of analytical performance, including linearity, limit of detection (LoD), limit of quantification (LoQ), and precision. For the assessment of interference, artificial plasma (BioChemazone) with two cfDNA samples isolated from single source healthy human donors (Ripple Biosolutions) were used at 1% dd-cfDNA.

Blood donor samples included 61 gDNA samples and 60 unique plasma samples from healthy volunteers. gDNA samples were prepared using Qiagen Blood Mini Kit (Qiagen), while plasma was isolated from whole blood using a double centrifugation protocol followed by cfDNA extraction using the QIAsymphony DSP Circulating DNA Kit (Qiagen). The blood donor samples were used to determine the limit of blank (LoB) and other analytical parameters.

### 2.2 Clinical samples

Clinical plasma samples were collected between 2022 and 2025. These samples included pre-transplant genomic DNA (gDNA) samples from the donor-recipient pairs and post-transplant plasma samples from the recipient used for relative dd-cfDNA level quantification. Blood was collected into Cell-Free BCT (Streck, Inc). Plasma was isolated using a two-step centrifugation protocol and stored at −18 °C until processing. Cell-free DNA (cfDNA) extraction was performed using the QIAsymphony DSP Circulating DNA Kit (Qiagen), with an elution volume of 45 μL. All cfDNA samples were analyzed for integrity (purity and concentration) using TapeStation Cell-free DNA ScreenTape (Agilent), according to the manufacturer’s instructions. The donor and recipient gDNA were extracted using the Qiagen Blood Mini Kit (Qiagen) according to the manufacturer’s instructions.

### 2.3 Marker selection and primer design

The TransplantTrace cfDNA Kidney assay employs 50 biallelic insertion–deletion (indel) markers selected for high heterozygosity (ability to differentiate between individuals), genomic stability (no known SNPs or sequence variants in the DNA sequence next to the targeted indel), and uniform chromosomal distribution. Candidate markers were evaluated using population frequency databases (i.e., 1,000 Genomes) and internal empirical testing to ensure minimal allele imbalance, minimal allelic amplification bias, low background noise, and consistent amplification. Markers were required to demonstrate robust performance across both gDNA and cfDNA contexts, including a high on-target percentage of the sequencing data (23).

### 2.4 Target amplification and sequencing

**Figure 1:**
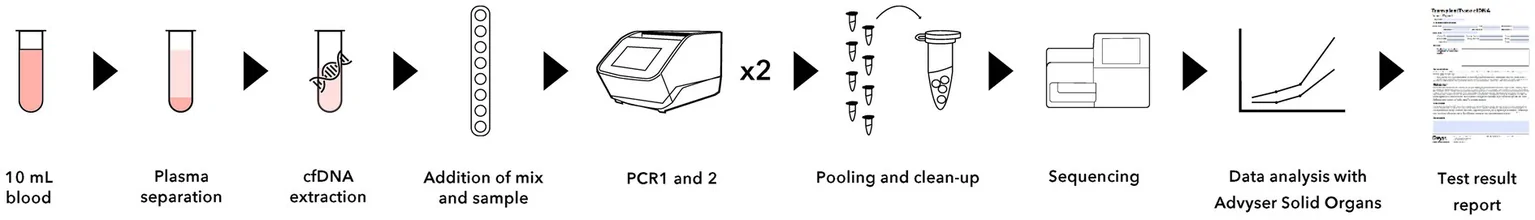
Schematic workflow for the TransplantTrace cfDNA Kidney assay.

Each sample underwent a two-step PCR protocol. In the first step (PCR1), marker-specific primers amplify the marker loci in a single multiplex reaction. In the second step (PCR2), unique sample-specific indices and adapters are added. Amplified libraries were pooled, purified using Devyser Library Clean reagents, and sequenced on the Illumina MiSeq platform using 2 × 75 bp paired-end reads. The resulting indel marker PCR products (amplicons) have an average size of 72 bp (including target specific primer) and a maximum size of 77 bp (with insertion and target specific primers), enabling almost full coverage using 2 × 75 cycles of sequencing. See Figure 1 for a schematic workflow.

### 2.5 Sequencing data analysis and sample quality control

Sequencing reads were demultiplexed, aligned to the human reference genome, and filtered for quality prior to analysis of the sample. Informative indel markers were defined as loci where donor and recipient genotypes differ in a way that permits quantification of the donor-derived allele. Samples were excluded if they failed to achieve a minimum required number of paired reads. The aimed minimum required number of reads was defined as 100 paired reads per pre-transplant sample and 5,000 paired-end reads per post-transplant sample to ensure adequate coverage depth of 10X for quantifying dd-cfDNA to a sensitivity of 0.2%. Sequencing data were analyzed to assess assay uniformity and on-target performance. For each sample, the proportion of reads aligning to targeted indel loci was calculated to evaluate on-target efficiency. Amplicon uniformity was assessed by measuring the sequencing depth across all samples and defined as the percentage of markers achieving the relative coverage of 40–160% of the mean read depth. Additionally, post-transplant samples were required to contain a minimum number of two informative markers to ensure reliable quantification. Analytical validity of pre-transplant genotyping, including identification of informative indel markers and validation of the ≥2-marker threshold was performed but is not included herein. Index read validation and quality control steps were applied to mitigate index hopping and misassignment.

### 2.6 Percent dd-cfDNA calculation

Donor and recipient genotype is required to enable detection of recipient and donor within a post-transplant plasma sample. The accompanying software, Advyser Solid Organs (Devyser AB), was used to determine the marker genotype as homozygote (i.e., +/+ or −/−) if the variant allele frequency (VAF) was between 0–1% or 99–100% and heterozygote (+/−) if the VAF is between 40 and 60%. To minimize noise, markers were excluded if the observed VAF was outside pre-defined genotype classification thresholds. The percentage of dd-cfDNA was calculated using the average allele frequency from all informative markers. For homozygous informative markers, the donor allele fraction was used directly. For heterozygous informative markers, the signal was adjusted by a factor of two. No background subtraction was applied, given the low sequencing error profile of indel-based design and short read lengths.

### 2.7 Analytical performance evaluation

Analytical performance was evaluated following applicable Clinical and Laboratory Standards Institute (CLSI) guidelines (e.g., EP05, EP06, EP07, EP17, and EP37).

#### 2.7.1 Analytical sensitivity

LoB was established using 180 blank samples measurements (i.e., a single genotype, pre-transplantation samples). LoB was defined as the highest measurement result that is likely to be observed within a stated probability [*α*] for a blank sample. The LoD was determined using 66 replicate samples prepared with a low amount of DNA from a second genotype, between 0.1 and 0.2%. LoD was defined as the lowest dd-cfDNA concentration that can be detected with a specified probability, corresponding to an acceptable false-negative rate. LoQ was verified using 36 low-level measurement samples at 0.3%. LoQ was defined as the lowest amount dd-cfDNA that can be quantitatively determined with acceptable accuracy and precision under stated experimental conditions. The theoretical LoD and LoQ were estimated using model-based analysis, whereas the experimentally verified LoD and LoQ correspond to the lowest concentration levels included in the discrete (stepwise) dilution series that met predefined detection criteria.

#### 2.7.2 Analytical precision

Precision was defined as closeness of agreement between indications or measured quantity values obtained by replicating measurements on the same or similar objects under specified conditions. This was assessed numerically using imprecision measures like coefficients of variation (CV) under specified conditions. The specified condition in this study was reproducibility (changed conditions, between-runs) and repeatability (unchanged conditions, within-run).

Reproducibility of the dd-cfDNA assay was evaluated in accordance with CLSI EP05 guidelines. The analysis incorporated multiple operators across different test series. Samples spanning target dd-cfDNA concentrations of 0.2, 1, and 30% were measured in replicates, resulting in a minimum of 30 total measurements per concentration. Statistical analyses included calculation of mean, standard deviation (SD), CV, and 95% confidence intervals (CI) for both means and SDs, to characterize assay precision under reproducibility conditions.

Repeatability (within-run precision) of the assay was evaluated according to CLSI EP05 guidelines. Measurements were performed on three target dd-cfDNA concentrations (0.2, 1, and 30%) using five replicate determinations each, all conducted by a single operator in one series. For each concentration, the mean, SD, and CV were calculated. 95% CI for the mean and SD were computed based on the Student’s t-distribution and Chi-square distribution, respectively. Statistical analysis was performed and included one-way ANOVA to confirm consistency across replicates.

#### 2.7.3 Analytical accuracy and linearity

The analytical accuracy and linearity of the TransplantTrace cfDNA Kidney assay were evaluated using both reference materials and clinical samples. Accuracy was assessed by comparing measured dd-cfDNA percentages to expected values from three independent dilution series ranging from 0.1 to 30%. Each series was tested in five replicates across two operators and reagent lots, resulting in a total of 270 measurements. Linearity was evaluated using linear regression analysis across the full range of concentrations. Additional stratified analyses were performed for lower (≤1%) and upper (>1%) concentration intervals to assess performance sensitivity across clinically relevant ranges.

To evaluate reproducibility and site-to-site agreement, clinical plasma samples from 22 kidney transplant recipients were analyzed. Each sample was independently tested at two clinical sites, yielding 44 paired measurements. Statistical analyses included linear regression, Pearson correlation, two-tailed paired t-test to assess differences between site results, and Bland–Altman analysis to evaluate agreement and potential bias between sites.

#### 2.7.4 Analytical specificity

To evaluate potential interference, selected endogenous (conjugated/unconjugated bilirubin, albumin, hemoglobin, triglycerides) and exogenous substances (anticoagulants, immunosuppressants including cyclosporine and mycophenolic acid) were spiked into artificial plasma containing cfDNA mimicking 1% dd-cfDNA. Each spiked sample underwent DNA extraction and analysis using the TransplantTrace cfDNA Kidney assay. Control samples were prepared in parallel under identical conditions but without the addition of any interfering substances. In total, 17 unique samples were prepared. Measured dd-cfDNA levels were compared to the 1% controls using statistical testing to evaluate the significance of any observed differences.

Cross-reactivity was defined as any response with similar DNA/RNA sequences that were not intended to be sequenced by the designed primers, and can lead to unspecific amplification, which then results in false-positive or false-negative results. Cross-reactivity was assessed through both *in-silico* analysis and empirical testing. For the *in-silico* analysis, each primer was analyzed using the National Center for Biotechnology Information (NCBI) Standard Nucleotide BLAST tool^1^ to identify potential off-target binding to human pseudogenes or non-human genomes. In addition, sequencing data from four clinical samples obtained from kidney transplant recipients were mapped to the human as well as the most common viral, bacterial, and fungal genomes to detect any non-specific amplification. Sequencing data from these analyses were processed through an in-house developed software pipeline. Reads not mapping to the targeted amplicons were flagged by the pipeline and used to determine the off-target percentage.

### 2.8 Clinical validation study

To assess clinical validity, 106 plasma samples were collected between 2022 and 2025 from kidney transplant recipients at the time of clinically indicated (for-cause) biopsy, and dd-cfDNA levels (%) were investigated. A clinically indicated (for-cause) biopsy was defined as a biopsy performed in response to suspected graft dysfunction based on clinical and/or biochemical findings, in accordance with the routine clinical practice at the site. In general, indications for biopsy included an unexplained increase in serum creatinine from baseline, decline in graft function, development or progression of proteinuria, and/or immunological findings such as the presence of donor-specific antibodies (including *de novo* DSA), as determined by the treating clinician. The time interval between biopsy and plasma collection was within 7 days (however samples collected within 48 h after biopsy were excluded to avoid potential biopsy-related elevations in dd-cfDNA). Genotyping of the recipient and donor was performed using gDNA. Two post-transplant samples were excluded from the analysis, because they did not fulfill the predefined study inclusion criteria. Both samples were collected within the immediate post-transplantation period (<14 days), a period during which clinical interpretation of dd-cfDNA testing is contraindicated in line with other assays. The remaining 104 samples were included in the final clinical validation study. Test operators were blind to biopsy and clinical information for all de-identified samples. For each patient, clinical variables such as gender at birth, age, time post-transplant, serum creatinine, and eGFR levels at the time of for-cause biopsy were collected and summarized. All biopsy specimens were reviewed by expert pathologists, and rejection status was classified according to the 2022 Banff Classification system. Formal independent re-evaluation and quantification of inter-rater agreement were not performed in this study. For the purpose of this study, the biopsies were classified as active rejection (AR) or no active rejection (non-AR). These groups were used for calculation of diagnostic performance metrics at a preselected cut-off of 1%, in line with predicate tests and literature. Subcategories of AR, including ABMR and TCMR, were reported but were not evaluated separately for performance. The results (% dd-cfDNA) reported using the TransplantTrace cfDNA Kidney test were combined with the clinical variables and biopsy-determined rejection status.

### 2.9 Statistical analysis

Statistical analyses of the analytical performance studies were as described in each relevant subsection. The objective of the statistical analysis for the clinical validation study was to determine whether the dd-cfDNA in a patient’s plasma can discriminate AR from non-AR allograft status in patients clinically indicated for biopsy. Analyses were performed retrospectively. No further stratification by rejection subtype was performed. Diagnostic performance of dd-cfDNA was assessed using sensitivity, specificity, and area under the curve (AUC). In addition, positive predictive value (PPV) and NPV were estimated at different prevalence (unadjusted, 15 and 25% prevalence). For comparative purposes, similar analyses were conducted for serum creatinine and eGFR to assess their ability to discriminate AR from non-AR. Pre-defined thresholds were applied for all reported performance parameters.

Open-source programming language R was used for all statistical analyses, and data visualization was performed with RStudio (v. 2025.05.1 + 513, Posit Software PBC). Statistical analyses included linear regression, Pearson correlation, two-tailed paired *t*-test, Bland–Altman analysis to evaluate agreement and potential bias between sites. Correlation and regression were calculated using Pearson’s least squares. Sensitivity and specificity were calculated using Jeffrey’s method.

## 3 Results

### 3.1 Sequencing metrics

On-target reads are sequencing reads that successfully align to the predefined genomic regions intended for analysis in a targeted NGS assay. The average on-target reads for all samples was 83.7%. Assay uniformity refers to the evenness of sequencing coverage across these targeted regions, indicating how consistently and reliably each region is captured and sequenced. The relative coverage of all samples was analyzed where 21,460 markers out of 24,400 markers had the relative coverage of 40–160% of mean read depth, resulting in 88% assay uniformity. These metrics were used to confirm consistent amplification, even coverage, and absence of substantial dropout across the 50-indel marker panel.

### 3.2 Analytical sensitivity

**Figure 2:**
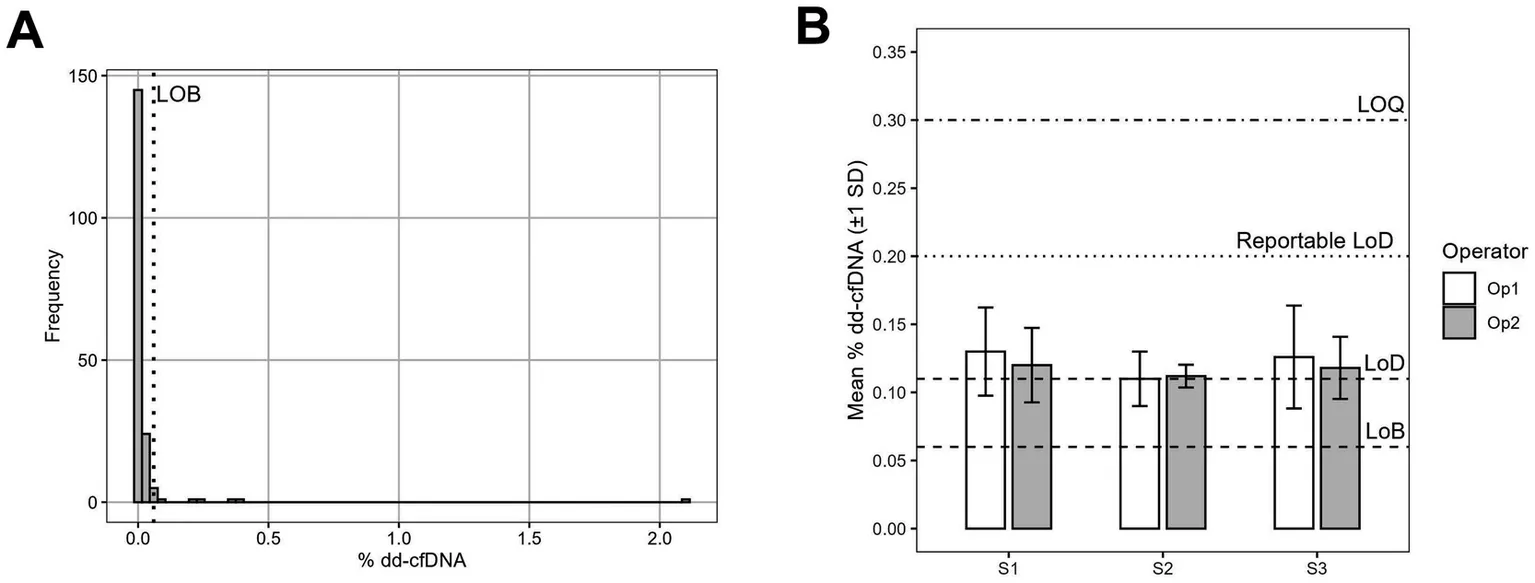
Analytical sensitivity of the TransplantTrace cfDNA Kidney assay. **(A)** Limit of blank (LoB) determined from 180 blank samples as the 95th percentile of the average background signal. **(B)** Limit of detection (LoD) determined using 66 replicate samples with low levels of cfDNA from a second genotype to estimate the probability of false-negative detection.

The analytical performance of the TransplantTrace cfDNA Kidney assay was assessed using sample mixtures with defined dd-cfDNA levels and blood samples from healthy donors. LoB was defined as the 95th percentile of the fraction of the average measured background in blank samples and was determined to be 0.06% (Figure 2A). Next, the LoD and LoQ of the assay were evaluated; where the LoD was determined to be 0.11% (Figure 2B) and verified at 0.2%; and the LoQ was verified at 0.3%. These findings indicate that the assay is capable of reliably detecting and quantifying low levels of dd-cfDNA in plasma samples.

### 3.3 Analytical precision

Reproducibility was assessed by analyzing 30 measurements per target concentration across multiple operators and series. The assay demonstrated robust reproducibility with mean values closely matching target concentrations: 0.21% (95% CI: 0.20–0.22) at 0.2, 1.11% (95% CI: 1.08–1.13) at 1, and 31.1% (95% CI: 30.7–31.5) at 30% dd-cfDNA. The observed standard deviations ranged from 0.031 at the lowest target to 1.06 at the highest, corresponding to CV of 14.7, 6.85, and 3.41%, respectively. These results confirm acceptable precision across the assay’s reportable range under reproducibility conditions (Table 1).

**Table 1:** Analytical precision parameters of the TransplantTrace cfDNA Kidney assay.

Analytical precision parameter	Target dd-cfDNA (%)	*n*	Mean (%)	95% CI for Mean	SD (%)	95% CI for SD	CV (%)
Reproducibility (between-run)	0.2	30	0.21	0.198–0.222	0.031	0.025–0.042	14.7
	1	30	1.11	1.08–1.13	0.076	0.060–0.102	6.85
	30	30	31.1	30.7–31.5	1.06	0.84–1.42	3.41
Repeatability (within-run)	0.2	5	0.20	0.18–0.22	0.017	0.010–0.050	8.7
	1	5	1.13	1.07–1.18	0.047	0.028–0.136	4.2
	30	5	29.7	29.3–30.2	0.39	0.23–1.12	1.3

dd-cfDNA, donor-derived cell-free DNA; CI, confidence interval; SD, standard deviation; CV, coefficient of variation.

Repeatability was assessed at dd-cfDNA target levels of 0.2, 1, and 30%, with five replicate measurements per concentration. The TransplantTrace cfDNA Kidney assay demonstrated consistent performance across all targets. The mean measured values closely approximated the target levels, with narrow 95% CIs (0.18–0.22% at 0.2%, 1.07–1.18% at 1%, and 29.3–30.2% at 30%). The within-run standard deviations were low, with corresponding 95% CI, indicating reliable precision (e.g., at 0.2% target, SD was 0.0173, 95% CI: 0.0104–0.0498%). The CV decreased with increasing concentration, ranging from 8.7% at 0.2 to 1.3% at 30%, demonstrating acceptable repeatability consistent with clinical requirements (Table 1).

### 3.4 Analytical accuracy and linearity

**Figure 3:**
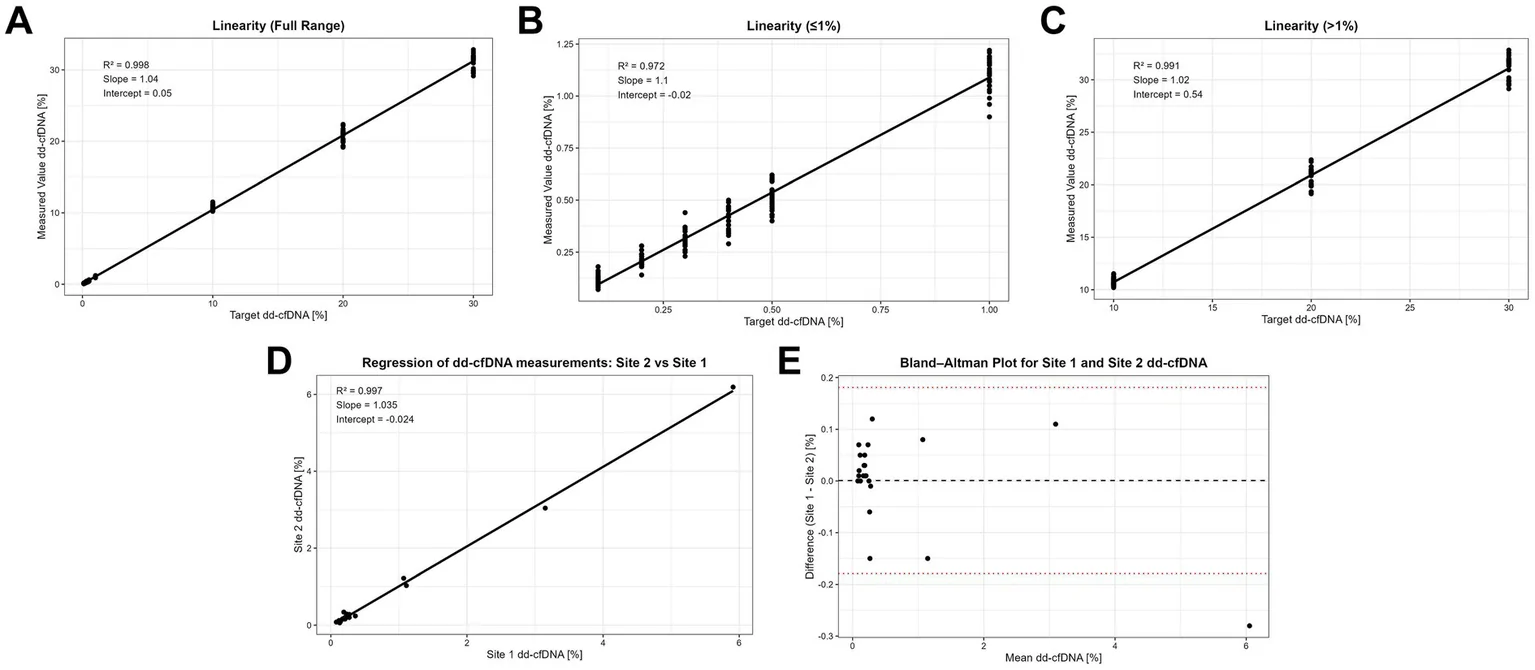
Analytical accuracy and linearity parameters of the TransplantTrace cfDNA Kidney assay. Measured donor-derived cell-free (dd-cf)DNA values were plotted against the expected target concentrations across three dilution series ranging nine levels between 0.1–30%. Linear regression was fitted to evaluate overall assay accuracy across **(A)** 0.1% and the full analytical range (0.2–30%); **(B)** in the low concentration range (0.1–1.0%); and **(C)** in the upper concentration range (10–30%). **(D)** Dd-cfDNA levels from 22 kidney transplant recipient plasma samples were independently measured at two sites. Results from Site 1 and Site 2 were compared using linear regression analysis. **(E)** Bland–Altman analysis showing the differences between dd-cfDNA measurements at Site 1 and Site 2 plotted against their mean. The dashed line represents the mean difference (bias), and dotted lines indicate the upper and lower limits of agreement.

The accuracy of the assay was established by comparing the measured percentage of dd-cfDNA results to the expected results from the reference materials. To assess the linearity of the dd-cfDNA assay within the range from 0.1 to 30%, a linear regression was fitted for three dilution series. The mean of slopes was 1.04, mean of y-intercepts was 0.05, and R^2^ value was 0.998 across all replicates (Figure 3A). These findings demonstrate a linear and accurate assay performance with minimal intercept deviation and strong agreement with theoretical values, supporting its high analytical trueness. To further enhance the assessment of analytical trueness, the linearity analysis was stratified into lower (≤1%) and upper (>1%) ranges (Figures 3B,C respectively). This approach enables a more sensitive evaluation of assay performance in clinically relevant low-percentage regions, where detection accuracy is particularly critical. In the lower range, the regression model demonstrated excellent linearity (*R*^2^ = 0.97), confirming the assay’s capability to reliably quantify low levels of dd-cfDNA with minimal bias. Similarly, the upper range maintained strong linearity (*R*^2^ = 0.991) and consistent slope values, underscoring the assay’s robustness across high levels. By evaluating these subranges independently, the assay’s precision and reliability are further validated across its full reportable range, supporting its use in both early rejection detection and high-level monitoring scenarios.

To further evaluate the performance of the assay, 22 clinical samples from kidney transplant recipients with dd-cfDNA percentages ranging from 0.1 to 6% were tested by two different testing sites. A high degree of linearity was observed between the two sites, with a correlation coefficient of 0.998 (95% CI: 0.996–0.999) and an *R*^2^ value of 0.997 (Figure 3D). Linear regression analysis showed a slope of 1.035 and an intercept of −0.024, indicating strong proportional agreement with minimal bias. A paired *t*-test revealed no statistically significant difference between the measurements from the two sites (*p* = 0.963). Furthermore, agreement analysis using a Bland–Altman plot demonstrated minimal bias and acceptable limits of agreement (Figure 3E), supporting the reproducibility of the assay across sites.

### 3.5 Analytical specificity

The TransplantTrace cfDNA Kidney assay demonstrated robust performance in interference studies. All tested substances and concentrations are provided in Supplementary Table S1. The presence of common endogenous (bilirubin, hemoglobin, triglycerides, albumin) and exogenous substances (cyclosporine, mycophenolic acid and EDTA) had no significant impact on assay performance, defined as allowable difference criterion ±15% compared to control sample. The highest tested concentration of unconjugated bilirubin (4 mg/dL) did not yield a result due to insufficient number of sequencing reads; however, all other evaluated concentrations (ranging 0.2–2 mg/dL) yielded results of stable assay performance.

To further assess potential off-target amplification due to pseudogenes or cross-reactivity with non-human DNA, both *in silico* and empirical analyses were conducted as described in the Materials and Methods. A total of 100 primers were subjected to BLAST analysis, which identified 30 alignments to viral, bacterial or fungal sequences. Importantly, none of these alignments had an overlapping primer pair (i.e., one forward and one reverse primer in the same, non-human species), indicating a low likelihood of off-target amplification. In addition, sequences generated with TransplantTrace cfDNA Kidney assay from clinical samples were mapped to the human genome as well as the most common microbial genomes. Less than 1% of the reads aligned to microbial genomes. These reads were subsequently removed by quality control filters in the analysis pipeline. As they did not map to target amplicons, these off-target sequences were not included in the final data output.

### 3.6 Assay input material

The concentration of circulating cfDNA can vary both between transplant recipients and within the same patient over time. Therefore, it is essential to assess assay performance across a range of input concentrations and define the minimum cfDNA input mass required for consistent, reliable results. A sample mixture with a dd-cfDNA level of 1% was serially diluted to assess performance across input concentrations ranging from 0.09375 to 3 ng/μL. Each concentration was tested in multiple replicates, and the precision and accuracy at each concentration level were assessed. The standard deviation and CV between samples remained low across most of the concentration range, demonstrating good reproducibility across varying input levels. The assay demonstrated consistent performance until the concentration of 0.1 ng/μL (corresponding to <2 ng input mass). When cfDNA input sample material was below this level (<0.1 ng/μL), a deterioration was observed in sequencing quality, i.e., % reads assigned to target declined, standard deviation between markers increased, and a minor loss in detection of dd-cfDNA, bias −0.11 (data not shown).

### 3.7 Increased dd-cfDNA correlates with biopsy-proven rejection in kidney transplant recipients

To confirm clinical performance of the assay, 104 plasma samples from kidney transplant recipients at the time of clinically indicated (for-cause) biopsy were collected and analyzed. Among the 104 paired biopsies, 23 were classified as AR, while the remaining 81 biopsies were classified as non-AR. Of the 23 AR, 12 were classified as ABMR and 11 were classified as TCMR. The non-AR cases included a number of pathological diagnostic categories such as interstitial fibrosis and tubular atrophy (IF/TA), calcineurin inhibitor (CNI) toxicity, glomerulonephritis, and cases without major findings. Patient characteristics of the clinical validation cohort are summarized in Table 2.

**Table 2:** Patient characteristics in the clinical validation study cohort.

Clinical characteristics	Active rejection (AR)	No active rejection (non-AR)
Number of samples (*n*)	23	81
Men (*n*, %)	12, 52.2%	48, 59.3%
Age at the time of sampling (years)	46.7	50.0
Post-transplant time (days)	1,564	1,088
Creatinine (μmol/L)	135.2	155.0
eGFR (mL/min)	59.9	47.2
dd-cfDNA (%)	1.2	0.2

Data is presented as mean values. eGFR, estimated glomerular filtration rate; dd-cfDNA, donor-derived cell-free DNA.

**Figure 4:**
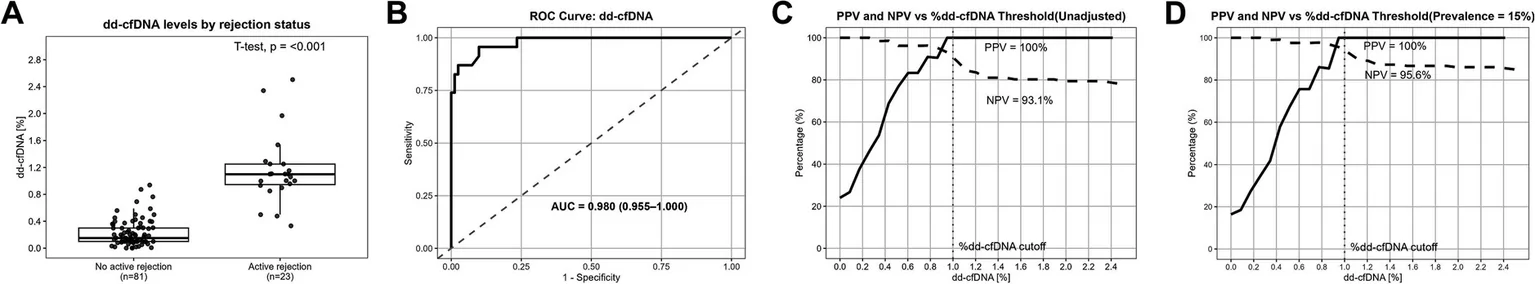
The TransplantTrace cfDNA Kidney assay distinguishes active rejection (AR) from non-active rejection (non-AR). **(A)** donor-derived cell-free (dd-cfDNA) fraction (%) in biopsy-confirmed AR (*n* = 23) and non-AR (*n* = 81). Box plots show medians and interquartile ranges. **(B)** receiver operating characteristic (ROC) curve for dd-cfDNA fraction discriminating AR from non-AR, with corresponding area under the curve (AUC). **(C,D)** positive predictive values (PPV; solid lines) and negative predictive values (NPV; dashed lines) for dd-cfDNA across different cut-offs, assuming **(C)** the observed (unadjusted) cohort prevalence and **(D)** an adjusted AR prevalence of 15%. The vertical dotted line indicates the selected dd-cfDNA cut-off at 1%, with corresponding PPV and NPV values annotated.

Plasma levels of dd-cfDNA (%) in recipients with biopsy-confirmed AR were determined and compared to percentage levels of dd-cfDNA in recipients without AR. The fraction of dd-cfDNA in plasma differed significantly between the groups (Figure 4A). The mean level of dd-cfDNA in patients with AR was significantly higher (1.2%) than in the comparator group (0.2%), without AR (*p* < 0.001). The AUC for distinguishing AR using dd-cfDNA was 0.980 (95% CI: 0.955–1.000), demonstrating excellent overall discriminatory ability of the assay (Figure 4B; Table 3).

**Table 3:** Diagnostic performance of dd-cfDNA, serum creatinine, and eGFR for detecting biopsy-proven active rejection (AR) in kidney transplant recipients.

Parameter		dd-cfDNA	Serum creatinine	eGFR
AUC		0.980 (0.955–1.000)	0.702 (0.576–0.828)	0.693 (0.573–0.814)
Threshold		1.0%	150 μmol/L	40 mL/min
Sensitivity		73.9% (53.9–88.3%)	13.6% (4.0–32.1%)	9.1% (1.9–26.1%)
Specificity		100% (97.0–100.0%)	67.1% (56.1–76.9%)	61.8% (50.6–72.2%)
Predictive values				
Unadjusted	PPV	100% (86.5–100.0%)	10.7% (3.1–25.9%)	6.5% (1.4–19.1%)
	NPV	93.1% (86.3–97.1%)	72.9% (61.7–82.2%)	70.1% (58.5–80.1%)
15% prevalence	PPV	100% (100.0–100.0%)	6.8% (0.0–14.0%)	4.0% (0.0–10.4%)
	NPV	95.6% (92.8–98.4%)	81.5% (77.8–84.7%)	79.4% (75.5–82.9%)
25% prevalence	PPV	100% (100.0–100.0%)	12.1% (0.0–25.5%)	7.4% (0.0–17.1%)
	NPV	92.0% (87.0–96.9%)	70.0% (65.0–75.2%)	67.1% (61.5–71.9%)

This table shows the AUC, diagnostic threshold (cut-off), sensitivity, specificity, and predictive values (PPV and NPV) for each marker with 95% CI. PPV and NPV are presented under three AR prevalence scenarios: No adjustment (study cohort), 15, and 25% prevalence. Abbreviations: dd-cfDNA, donor-derived cell-free DNA; eGFR, estimated glomerular filtration rate; AUC, area under the curve; PPV, positive predictive value; NPV, negative predictive value.

With a pre-selected cut-off of 1.0%, the dd-cfDNA test demonstrated 100% specificity (95% CI: 97.0–100%) and 73.9% sensitivity (95% CI: 53.9–88.3%). Without prevalence adjustment, the PPV was 100% (95% CI: 86.5–100%) and the NPV was 93.1% (95% CI: 86.3–97.1%) (Figure 4C; Table 3). PPV and NPV across different assumed prevalences of rejection are shown in Figure 4D (adjusted to 15% prevalence) and Supplementary Figure S1A (adjusted to 25% prevalence).

**Figure 5:**
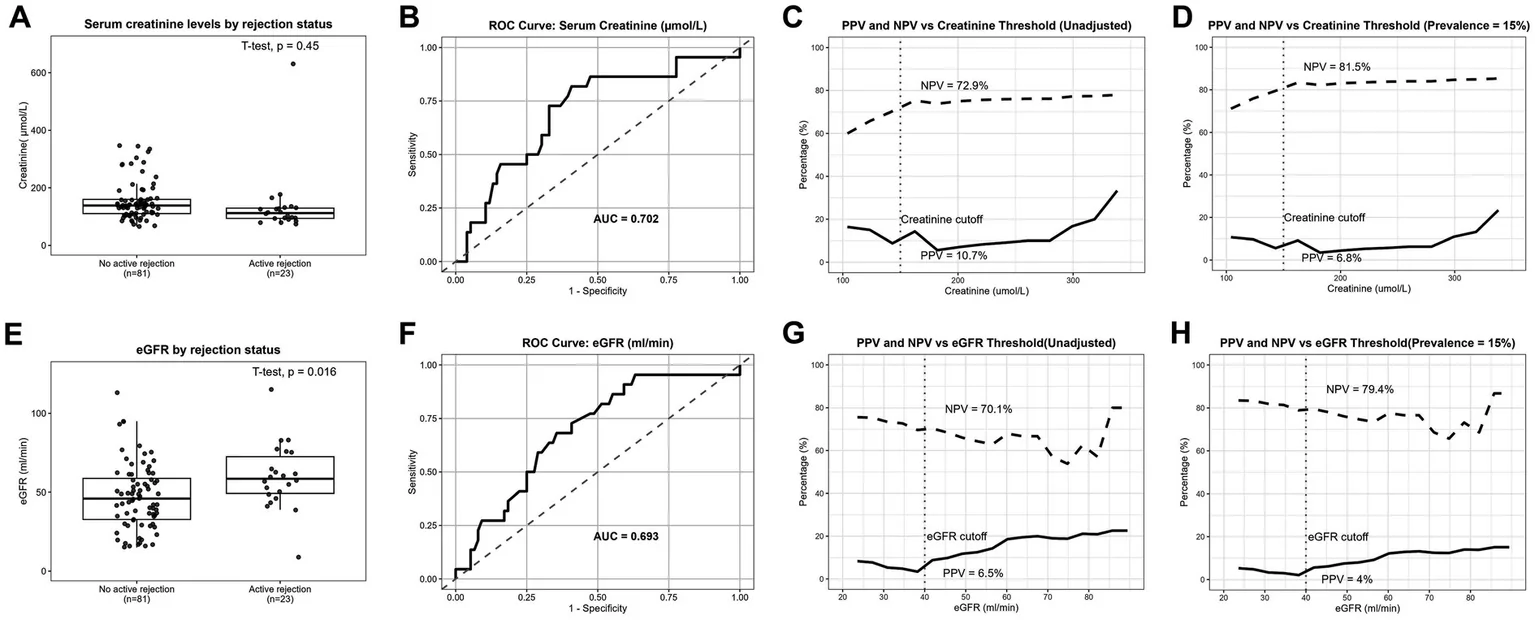
Serum creatinine and *eGFR* show limited ability to discriminate active rejection. **(A)** Serum creatinine levels (μmol/L) in patients with biopsy-proven active rejection (AR, *n* = 23) compared to those without active rejection (non-AR, *n* = 81). Boxplots show medians and interquartile ranges. **(B)** Receiver operating characteristic (ROC) curve and area under the curve (*AUC*) for serum creatinine to discriminate AR from non-AR. **(C,D)** Positive predictive values (*PPV*; solid lines) and negative predictive values (*NPV*; dashed lines) for serum creatinine across different cut-offs, assuming **(C)** the observed (unadjusted) cohort prevalence and **(D)** an adjusted AR prevalence of 15%. **(E–H)** Corresponding analysis for estimated glomerular filtration rate (*eGFR*, mL/min), including **(E)** group comparison, **(F)** ROC curve with *AUC*, and *PPV*/*NPV* plots assuming **(G)** observed prevalence and **(H)** 15% adjusted prevalence. Vertical dotted lines indicate selected cut-offs (150 μmol/L for serum creatinine and 40 mL/min for *eGFR*), with corresponding *PPV* and *NPV* values annotated.

In comparison, levels of serum creatinine and eGFR at the time of biopsy were less reliable (Figure 5; Table 3). The mean level of serum creatinine in patients with AR was 135.2 μmol/L and 155 μmol/L in the group without AR (*p* = 0.45) (Figure 5A). The ROC curve for serum creatinine to discriminate AR from non-AR had an AUC of 0.702 (95% CI: 0.576–0.828) at any cut-off level for creatinine (Figure 5B), while unadjusted PPV was 10.7% and NPV was 72.9% (Figure 5C). PPV and NPV adjusted to 15 and 25% prevalence are shown in Figure 5D and Supplementary Figure S1B, respectively. Mean level of eGFR in patients with AR was 59.9 mL/min and 47.2 mL/min in the group without AR (*p* = 0.016) (Figure 5E). The ROC curve for eGFR to discriminate AR from non-AR had an AUC of 0.693 (95% CI: 0.573–0.814) (Figure 5F), while unadjusted PPV was 6.5% and NPV was 70.1% (Figure 5G). PPV and NPV with prevalence adjusted to 15 and 25% are presented in Figure 5H and Supplementary Figure S1C, respectively. Taken together, serum creatinine and eGFR showed limited ability to discriminate active rejection from non-active rejection and performed less effectively than fractional dd-cfDNA measured by TransplantTrace cfDNA Kidney.

## 4 Discussion

The number of patients undergoing kidney transplantation is increasing, with a new record of more than 28,000 conducted transplantations in the United States alone in 2023 (3). Today, post-transplantation care is focused on maintaining long-term allograft function, preventing immune-mediated injury, and minimizing adverse effects from immunosuppressive therapy. Traditional monitoring strategies, including serum creatinine and invasive renal biopsy have limitations and warrant for improved non-invasive strategies to monitor allograft status. As transplantation volumes rise, the need for non-invasive tools capable of detecting early signs of allograft injury has become increasingly important to improve long-term graft outcomes. Dd-cfDNA has emerged as a non-invasive clinically relevant biomarker which enables monitoring of graft integrity and may support earlier intervention. Various thresholds for dd-cfDNA fractions have been proposed and used in the literature (13, 24), and a commonly referenced cut-off for detecting active rejection in kidney transplant recipients is 1.0% (11, 12, 25). However, the optimal threshold may vary depending on numerous factors and remains a topic of further studies, as also observed in this study. To date, several centralized platforms for the detection of dd-cfDNA have been validated but there is a need for continuous data generation in the field.

In this study, the TransplantTrace cfDNA Kidney assay demonstrated high analytical precision, strong linearity, and no interference from tested substances, supporting its robust analytical validity. At the commonly applied dd-cfDNA threshold of 1.0%, the assay demonstrated an AUC of 0.980, sensitivity of 73.9% and specificity of 100%, enabling timely intervention in cases of active rejection while potentially reducing the need for unnecessary biopsies in patients with stable graft function. The similarly sized prospective DART study by Bloom et al. (*n* = 102 patients) reported an AUC of 0.74, sensitivity of 59.3% and specificity of 84.7%. The study included 27 active rejection cases and 75 no-rejection cases, with rejection status determined based on for-cause biopsy findings (11). However, direct comparisons across studies should be interpreted with caution due to differences in, e.g., study design, cohort composition, diagnostic criteria, assay methodology and more. In line with previous studies (4, 11, 12), serum creatinine failed to adequately discriminate AR from non-AR, underscoring the limitations of conventional kidney function biomarkers, as reflected by an AUC of 0.702 and a low PPV (10.7%). In contrast, the fraction of dd-cfDNA was significantly elevated in biopsy-confirmed AR compared to non-AR, supporting the assay’s robust clinical validity. These findings are the first report on this centralized assay and support its implementation as a competitive assay with excellent performance and favorable characteristics, in clinical settings where donor genotyping can be obtained. At the same time, while the selected commonly used 1.0% threshold prioritizes maximal specificity and may be well suited for ruling out rejection and avoiding unnecessary biopsies, future data-driven analysis of lower cut-offs may increase sensitivity and may support earlier detection in higher-risk settings.

There are several limitations with this study. The number of biopsy-confirmed active rejection cases was limited (*n* = 23) and all estimates should be interpreted with consideration of the corresponding confidence intervals, which reflect the uncertainty associated with the sample size. The number of AR cases, was considered adequate and appropriate for evaluating the assay’s ability to discriminate AR from non-AR, and was in line with the sample size as reported by, e.g., Bloom et al. (11). Although subgroups of AR (ABMR and TCMR) were reported, separate calculations of performance were not included. In addition, while the utility of dd-cfDNA testing for longitudinal trend monitoring post-transplantation has been demonstrated in multiple pivotal studies, this aspect was not evaluated in the present study and warrants further investigation, ideally using a prospective study design. Another limitation of this study is that the cohort consisted exclusively of a for-cause biopsy population enriched for patients with clinically suspected graft dysfunction and the results may therefore not apply to routine screening settings. As the prevalence of rejection is higher in for-cause settings than in routine surveillance settings, this may influence the observed predictive values. In a lower-prevalence surveillance setting, these predictive values would be expected to differ, and the provided prevalence-adjusted estimates are intended to illustrate this variation across risk settings. As predictive values are inherently dependent on disease prevalence, their application to routine surveillance populations should be interpreted in light of the underlying likelihood of rejection in the tested population. In contrast, sensitivity and specificity are less influenced by cohort composition and more directly reflect the intrinsic performance of the assay. Future studies in unselected surveillance populations can further define clinical utility across the full spectrum of transplant care.

Despite these limitations, the performed clinical validation, together with the extensive analytical validation of the assay, corroborates its use for determination of dd-cfDNA, building on the added value and utility of this marker demonstrated by other studies, e.g., (14) among others.

In summary, the data presented in this study validates the use of the TransplantTrace cfDNA Kidney assay to detect dd-cfDNA in kidney recipients. Dd-cfDNA has emerged as a reliable, non-invasive biomarker for detecting early graft injury and monitoring the status of transplanted organs. By identifying early signs of rejection, dd-cfDNA testing enables clinicians to initiate timely and proactive treatment interventions, ultimately supporting improved transplant outcomes. By integrating dd-cfDNA testing into routine post-transplant care, clinicians can improve diagnostic precision, personalize treatment strategies, reduce unnecessary biopsies, and ultimately maximize long-term allograft survival. TransplantTrace cfDNA Kidney enables detection of dd-cfDNA with short turnaround time, with high sensitivity and using low input DNA, providing a robust and competitive alternative to currently available centralized dd-cfDNA testing solutions.

## Data Availability

The raw data supporting the conclusions of this article will be made available by the authors, without undue reservation.

## Footnotes

1
https://blast.ncbi.nlm.nih.gov/Blast.cgi
