# Supplementary material for: Validation of the TransplantTrace cfDNA Kidney assay for measurement of donor-derived cell-free DNA in transplant recipients
Source: Front Med (Lausanne). 2026 Aug 18;13:1880342. doi: 10.3389/fmed.2026.1880342 (PMC13529464; doi:10.3389/fmed.2026.1880342)
Supplement: Supplementary file 1 [file Supplementary_file_1.DOCX]

Supplementary Material

**Supplementary Figure 1.** Positive predictive value (PPV; solid line) and negative predictive value (NPV; dashed line) assuming 25% active rejection (AR) prevalence shown for selected threshold (cut-off at vertical dashed line): (**A**) dd-cfDNA (%) measured with TransplantTrace cfDNA Kidney at a cut-off at 1%; (**B**) serum creatinine at a cut-off at 150 µmol/L; and (**C**) estimated glomerular filtration rate (eGFR) at a cut-off at 40 mL/min.

**Supplementary Table 1**. Tested interfering substances and their concentrations.

| **Interfering substance** | **Tested concentrations (g/mL)** |
| --- | --- |
| Albumin | 0.06 |
| Hemoglobin | 0.0001 |
| Bilirubin, conjugated | 0.0004 |
| Bilirubin, unconjugated | 0.000002, 0.000004, 0.000008, 0.00001, 0.00002, 0.00004 |
| Triglycerides | 0.015 |
| EDTA | 0.00114 |
| Mycophenolic acid | 0.000042 |
| NaOH | 0.002 M |
| Cyclosporine | 0.0000018 |
